# Environmental quality affects the formation of generalist and specialist taxa in microbial communities

**DOI:** 10.1093/ismeco/ycag057

**Published:** 2026-03-17

**Authors:** Guo Yang, Feng Wen, Dianfu Wang, Didi Zhao, Qin Liu, Min Ren, Jinshui Zheng, Ruili Zhang, Zhanfeng Xia, Jing Zhang, Lili Zhang, Chuanxing Wan, Xiaoxia Luo

**Affiliations:** State Key Laboratory Incubation Base for Conservation and Utilization of Tarim Basin, Alar, Xinjiang 843300, China; College of Life Science and Technology, Tarim University, Alar, Xinjiang 843300, China; State Key Laboratory Incubation Base for Conservation and Utilization of Tarim Basin, Alar, Xinjiang 843300, China; College of Life Science and Technology, Tarim University, Alar, Xinjiang 843300, China; State Key Laboratory Incubation Base for Conservation and Utilization of Tarim Basin, Alar, Xinjiang 843300, China; College of Life Science and Technology, Tarim University, Alar, Xinjiang 843300, China; State Key Laboratory Incubation Base for Conservation and Utilization of Tarim Basin, Alar, Xinjiang 843300, China; College of Life Science and Technology, Tarim University, Alar, Xinjiang 843300, China; State Key Laboratory Incubation Base for Conservation and Utilization of Tarim Basin, Alar, Xinjiang 843300, China; College of Life Science and Technology, Tarim University, Alar, Xinjiang 843300, China; State Key Laboratory Incubation Base for Conservation and Utilization of Tarim Basin, Alar, Xinjiang 843300, China; College of Life Science and Technology, Tarim University, Alar, Xinjiang 843300, China; National Key Laboratory of Agricultural Microbiology, Huazhong Agricultural University, Wuhan 430070, China; College of Food Science and Engineering, Tarim University, Alar, Xinjiang 843300, China; State Key Laboratory Incubation Base for Conservation and Utilization of Tarim Basin, Alar, Xinjiang 843300, China; College of Life Science and Technology, Tarim University, Alar, Xinjiang 843300, China; State Key Laboratory Incubation Base for Conservation and Utilization of Tarim Basin, Alar, Xinjiang 843300, China; College of Life Science and Technology, Tarim University, Alar, Xinjiang 843300, China; State Key Laboratory Incubation Base for Conservation and Utilization of Tarim Basin, Alar, Xinjiang 843300, China; College of Life Science and Technology, Tarim University, Alar, Xinjiang 843300, China; State Key Laboratory Incubation Base for Conservation and Utilization of Tarim Basin, Alar, Xinjiang 843300, China; College of Life Science and Technology, Tarim University, Alar, Xinjiang 843300, China; State Key Laboratory Incubation Base for Conservation and Utilization of Tarim Basin, Alar, Xinjiang 843300, China; College of Life Science and Technology, Tarim University, Alar, Xinjiang 843300, China

**Keywords:** Tarim River Basin, ecological index, community assembly

## Abstract

Microorganisms play a central role in global biogeochemical cycles, and generalists and specialists differ significantly in environmental adaptability, ecological functions, and community stability. However, current studies on generalists and specialists have mainly focused on habitat type or resource availability, while how habitat quality affects their geographic distribution and succession processes remains unclear. This study investigates how habitat quality influences the geographical distribution and community assembly of generalists and specialists. Using the Remote Sensing Ecological Index (RSEI), we assessed soil ecological quality in the Tarim River Basin and categorized the habitats into low-quality (extreme poor), moderate-quality (moderate poor), and high-quality (relatively good) zones. We analyzed the geographical patterns of generalists/specialists and their community assembly processes as habitat quality declined. Results indicated significant differences in microbial community composition (β-diversity, Bray–Curtis distance) between generalist and specialist groups across habitats. As RSEI decreased, the relative abundance of specialist microorganisms increased, while generalist microorganisms decreased. Microbial community assembly was shaped by both stochastic and deterministic processes, with stochastic processes accounting for >75%. Deterministic processes have a greater impact on specialists than generalists. This study provides insights into how environmental changes affect microbial ecosystems and their dynamics.

## Introduction

Microbial communities have always been central to soil nutrient cycling and ecosystem functioning; recent shifts in ecosystem quality driven by extreme events such as heat waves, sandstorms, and altered precipitation patterns [1] are now changing the composition and activity of these microbes, with cascading effects on soil productivity and other ecosystem processes [2]. Although microbial community composition and assembly have been extensively studied within individual ecosystems, the impact of ecosystem quality degradation on these communities has received far less attention and remains poorly understood [3–5]. Nevertheless, in-depth research on the effects of ecosystem quality degradation on microbial communities, particularly their survival strategies under such stress, provides new perspectives for understanding how environmental changes drive the ecological and evolutionary dynamics of microbial communities.

Remote-sensing technology has proven highly effective for acquiring spatiotemporal datasets of key land-surface parameters such as spectral reflectance, vegetation indices (e.g. NDVI), soil moisture, and surface temperature across vast regions, making it an invaluable tool for rapid detection of environmental changes at the regional scale [6]. Since its introduction by Xu in 2013, the Remote Sensing Ecological Index (RSEI) has proven a highly efficient tool for data collection, eliminating the need for human input in weighting, and providing an objective reflection of ecosystem quality, defined as the integrated state of vegetation coverage, humidity, temperature conditions, and land use intensity within an ecosystem [7]. The RSEI has been widely used to assess ecosystem quality in various settings such as watersheds [8], plateau [9], urban [10], and continental scales [11]. Although ecosystem quality has been extensively characterized across temporal and spatial scales, its impact on microbial communities has received little attention [7]. Microbial communities are vital components of ecosystems, and their abundance and function play crucial roles in maintaining ecosystem stability and driving biogeochemical cycles. Microbial communities consist of diverse microorganisms, each occupying a distinct niche, allowing them to be classified as either habitat generalists or specialists [12]. Habitat generalists are remarkably ubiquitous, with wide habitat preferences, whereas specialists are specific to certain habitats with narrow environmental tolerances [13]. Owing to differences in environmental adaptability, habitat generalists and specialists are expected to have distinct assembly patterns. However, there is limited knowledge on how ecosystem quality influences the assembly patterns of these groups.

The assembly of microbial communities involves interspecific relationships, environmental selection, and stochastic events that collectively determine the structure and function of the community [14]. Niche theory and neutral theory are two core frameworks for understanding microbial community assembly. According to niche theory, microbial communities are shaped by deterministic processes driven by local environmental factors and biological interactions [15]. In contrast, the neutral theory posits that stochastic processes (birth, death, mutation, and limited dispersal) govern the assembly of microbial communities [16, 17]. Furthermore, increasing evidence suggests that both stochastic and deterministic processes play regulatory roles in microbial community assembly [18]. However, most existing studies have focused on investigating the effects of the ecosystem type on microbial communities as a whole, whereas research on how the decline in habitat quality influences the community assembly patterns of habitat generalists and specialists remains limited [19–21]. Therefore, in-depth research on the effects of habitat quality on habitat generalists and specialists, particularly on the assembly patterns and adaptive mechanisms of species with different abundance characteristics, is crucial for understanding how ecosystems regulate habitat generalists and specialists.

The Tarim River Basin, one of the world’s largest inland river basins, exerts significant influence on the global climate and water cycle [22]. However, human activities and excessive water consumption have deteriorated the environmental quality of the Tarim River Basin [8]. Despite being the largest inland river in China, Tarim River has been the focus of relatively few studies on microbial diversity, particularly when compared with the extensive research conducted on major basins such as the Yangtze, Danube, and Pearl Rivers [23, 24]. Previous studies in the Tarim River Basin have investigated microbial communities associated with specific plant species in the lower reaches such as *Populus euphratica* and *Tamarix* and revealed shifts in fungal and bacterial diversity related to plant development and soil function [25, 26]. However, these studies were limited to localized habitats and did not assess microbial community assembly mechanisms or broader spatial patterns across multiple habitat types, which our study addresses at the scale of the entire river system.

Based on the ecological quality map of the Tarim River Basin generated using the Remote Sensing Ecological Index (RSEI), which visualizes habitat conditions across the region (Fig. S1), this study aimed to address the following questions using 16S rRNA and ITS amplicon sequencing: (i) distinguish the impact of ecosystem quality on generalists and specialists in the Tarim River Basin, and (ii) distinguish the assembly patterns of generalists and specialists in the Tarim River Basin and elucidate the effects of environmental factors on generalists and specialists. We hypothesized that ecosystem quality is a key environmental gradient influencing both generalists and specialists, but that their community assembly patterns respond differently being predominantly shaped by stochastic processes in generalists and deterministic processes in specialists, depending on ecosystem conditions.

## Materials and methods

### Study area and methods overview

We surveyed 227 composite soil samples (10–20 cm) across the Tarim River Basin, Xinjiang, China (34.2–43.39° N, 71.39–93.45° E) during July–August 2022. Habitat quality at each site was quantified using the RSEI derived from normalized difference vegetation index (NDVI), land surface moisture (Wet), land surface temperature (LST), and normalized differential built-up and bare soil index (NDBSI), following HJ/T 192–2015 and consolidated into three categories: extreme poor (RSEI <0.20; *n* = 99), moderate poor (0.20–0.35; *n* = 59), and relatively good (0.35–0.55; *n* = 69) (full workflow and site metadata in our previous study [27]; Fig. S1). Within each 50 × 50 m plot, three cores were composited (~500 g) and stored cold. Soil physicochemistry [total nitrogen (TN), total phosphorus (TP), total potassium (TK), ammonium nitrogen (NH_4_-N), nitrate nitrogen (NO_3_-N), total organic carbon (TOC), pH, soil electrical conductivity (EC), soil organic phosphorus (SOP), inorganic P, water-soluble organic carbon (DOC) and water-soluble organic nitrogen (DON)] was measured using standard protocols; mean annual temperature (MAT) and mean annual precipitation (MAP) were extracted from WorldClim v2 (30″). DNA was extracted (Omega), 16S V3–V4 (338F/806R) and fungal ITS1 (ITS5/ITS2) were amplified, and libraries sequenced on Illumina NovaSeq PE250. Reads were processed with USEARCH/VSEARCH (primer trimming, dereplication, chimera filtering) and UNOISE3 to infer ZOTUs (97% OTUs additionally generated where required); taxonomy was assigned using RDP (version 11.5; bacteria) and UNITE (version 7.1; fungi). Detailed parameters, QC thresholds, and sequence counts are provided in Supplementary Methods.

### Analysis of habitat generalists and specialists

To classify species as generalists, specialists, or neutrals, we considered the species frequency (Levins’ niche width) in the observed community matrix across the RSEI gradient compared to permutated matrices followed a null model approach [28]. Specifically, we generated 1000 permuted community matrices using the function spec.gen() implemented in the “EcolUtils” R package (https://github.com/GuillemSalazar/EcolUtils), which randomly reshuffles species occurrences across samples while maintaining the total species richness and sample richness (i.e. row and column sums of the presence–absence matrix). For each species, the frequency of occurrence (number of samples in which the species occurs) was calculated for both the observed and permuted matrices using Levins’ niche width index by “spaa” R package. The null distribution of occurrence frequencies from the 1000 permutations was used to calculate the 95% confidence interval for each species. Species whose observed frequency exceeded the upper limit of this 95% CI were classified as generalists (significantly more widespread than expected by chance), those below the lower limit were classified as specialists (significantly more restricted), and the remaining species were considered neutral. The Levins’ niche width index was used to quantify the ecological niche breadth of each species based on its distribution across environmental gradients. This index reflects habitat specialization (i.e. resource or environmental range use), whereas the permutation-based classification from EcolUtils identifies species that are significantly more or less widespread than expected by chance. Thus, species classified as “specialists” under the null model are not simply rare, but those whose restricted distribution is statistically narrower than random expectation, consistent with low niche breadth values.

### Statistical analysis

This study investigated the proportions of stochastic and deterministic processes in microbial communities in different environments in the Tarim River Basin. The “trans_beta” function in the “microeco” package was used to conduct principal coordinate analysis (PCoA) based on the Bray–Curtis distance metric to assess β diversity, while the Wilcoxon test was used to assess between-group distance differences. The “trans_abund” function of the “microeco” package in R [29] was used to calculate the relative abundance of specific taxa, whereas the “vegan” package was used to identify ZOTUs that are distinct and shared across the three habitat levels. This study used the Similarity Percentages (SIMPER) test in the “vegan” package to assess the contribution rate of each ZOTU to bacterial community differences. The top 250 species contributing to the differences were phylogenetically analyzed using the ML method and visualized using the iTOL online platform (https://itol.embl.de/). Pearson rank correlations were calculated between environmental variables and the relative abundances of the top 250 differential OTUs identified by SIMPER analysis. The resulting *P*-values were adjusted for multiple testing using the Benjamini–Hochberg false discovery rate (FDR) correction, and only correlations with adjusted *P* < .05 were retained for identify the effects of significantly different environmental and climatic factors on the microbial communities.

To determine the potential importance of stochastic processes in community assembly, the “MicEco” package (https://github.com/Russel88/MicEco) was used to calculate a neutral community model for predicting the microbial community formation process. The Sloan neutral model was used to evaluate the effects of stochastic dispersal and ecological drift on the assembly of microbial communities in the Tarim River Basin. Generally, the model predicts that taxa abundant in the metacommunity are likely to be widely distributed because they are more likely to disperse by chance among different sampling sites, whereas rare taxa are more likely to be lost at different sites owing to ecological drift (stochastic loss and replacement of individuals). This model fits the occurrence frequency of microorganism ZOTUs and their abundance across the metacommunity, with a single free parameter describing the migration rate “m” of individual microorganism ZOTUs [30]. The estimated “m” represents the probability that a random loss of an individual taxon from a local community will be replaced by dispersal from the metacommunity, and can thus be interpreted as a measure of dispersal limitation. A higher “m” value indicates less dispersal-limited communities. The *R*^2^ parameter indicates the goodness of fit to the neutral model.

Representative ZOTUs were aligned using MUSCLE v5.1 (default settings), and a maximum-likelihood phylogenetic tree was constructed with FastTree v2.1.11 under the GTR model, serving as the backbone for iCAMP analysis. Community assembly mechanisms were inferred using the iCAMP framework, a phylogenetic bin-based null model implemented via the open-source R package “iCAMP” (CRAN) and custom scripts from GitHub (https://github.com/DaliangNing/iCAMP1). Five ecological processes were quantified across microbial bins: homogeneous selection (HoS), heterogeneous selection (HeS), dispersal limitation (DL), homogenizing dispersal (HD), and drift (DR) [20, 31]. All analyses were conducted in R 4.3.1, and maps were produced in ArcGIS 10.5 (ESRI).

## Results

### Changes in community composition of generalist and specialist populations under varying habitat qualities

**Figure 1:**
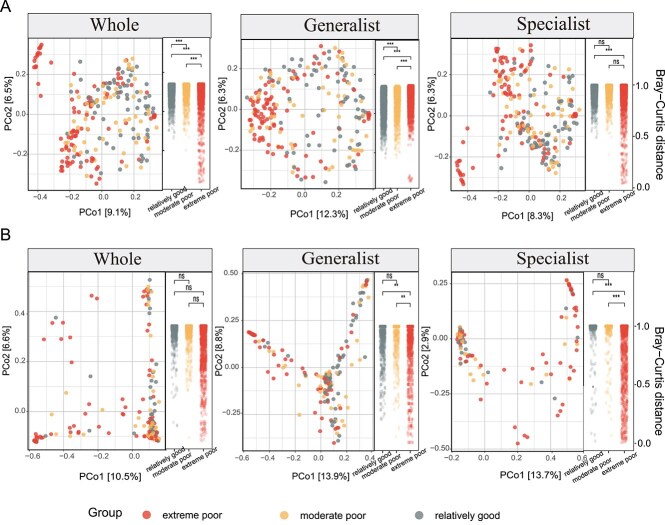
Diversity of microbial communities in different habitats in the Tarim River Basin. PCoA on the basis of Bray–Curtis distance and intergroup distance differences of (A) bacteria and (B) fungi. The hierarchical tree on the right side of the PCoA chart shows the differences in distances between groups in different habitats. ^*^: *P* < .05, ^**^: *P* < .01, ^***^: *P* < .001. Ns: *P* > .05.

The PCoA results revealed a notable influence of habitat quality on the overall bacterial community composition and clear separation between the generalist and specialist groups (*P* < .001) (Fig. 1A). Although the whole fungal community did not show significant differences across the three habitat qualities, a highly significant difference was observed between the generalist and specialist fungal communities (*P* < .01) (Fig. 1B). Pairwise Bray–Curtis distances within and among groups are explicitly illustrated as scatter plots on the right side of each PCoA panel, clearly showing variations in community similarity across habitat groups. Linear regression analyses showed contrasting responses of generalist and specialist taxa to RSEI (Fig. S2). In bacteria, generalists increased while specialists decreased with higher RSEI, indicating a shift toward ecological generalization under improved environmental conditions (Fig. S2A). In fungi, both trends were weaker, suggesting a lower sensitivity of fungal communities to habitat quality gradients (Fig. S2B). Generalist bacteria exhibited higher occupancy breadth than specialists across environmental types, while specialists showed stronger habitat specificity, as indicated by most FDR-significant indicators (Fig. S3A–C). In contrast, fungal occupancy differences were minimal, with no significant habitat indicators identified (Fig. S3D and E).

**Figure 2:**
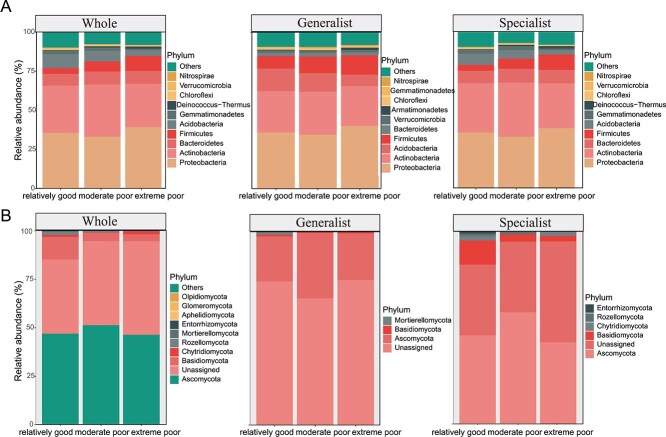
Phylum-level composition of the whole microbiome and of generalist vs specialist taxa across habitat qualities. Only the top 10 dominant species in terms of relative abundance are shown: (A) Bacteria; (B) Fungi.

Taxonomic analysis revealed that *Proteobacteria*, *Actinobacteria*, *Bacteroidetes*, *Firmicutes*, and *Acidobacteria* were the predominant bacterial phyla, collectively representing 35.86%, 30.26%, 8.07%, 6.53%, and 6.46% of the community, respectively (Fig. 2). In the generalist communities, *Proteobacteria* (average of 36.97%) and *Firmicutes* (average of 10.3%) were more abundant than in the overall bacterial community. Moreover, compared with the entire bacterial community and the generalist community, the specialist community exhibited greater abundances of *Actinobacteria* (31.6%) and *Bacteroidetes* (8.43%). The generalist community lacked the *Deinococcus–Thermus* species (Fig. 2A). *Ascomycota*, *Basidiomycota*, and *Chytridiomycota* were the predominant fungal phyla, representing 48.06%, 6.5%, and 0.4% of the community, respectively (Fig. 2B). In the specialist community, *Ascomycota* (48.53%) and *Chytridiomycota* (2.03%) were more abundant than in the bacterial and generalist communities (Fig. 2B). We used Venn diagrams (Fig. S4) to examine ZOTU overlap between relatively good, moderate poor, and extreme poor habitats. Bacterial generalists (Fig. S4A) and fungal generalists (Fig. S4C) showed high overlap across all habitats, with 100% and 96.2% of ZOTUs shared, respectively.

In contrast, specialists were more habitat-specific: for bacteria (Fig. S4B), 95% of ZOTUs were shared, but many were unique to extreme poor (77) or relatively good (68) habitats. Fungal specialists (Fig. S4D) were more habitat-specific, with 39.7% of ZOTUs shared across all environments and the rest restricted to one or two habitat types.

### Relative influence of stochastic vs deterministic processes

**Figure 3:**
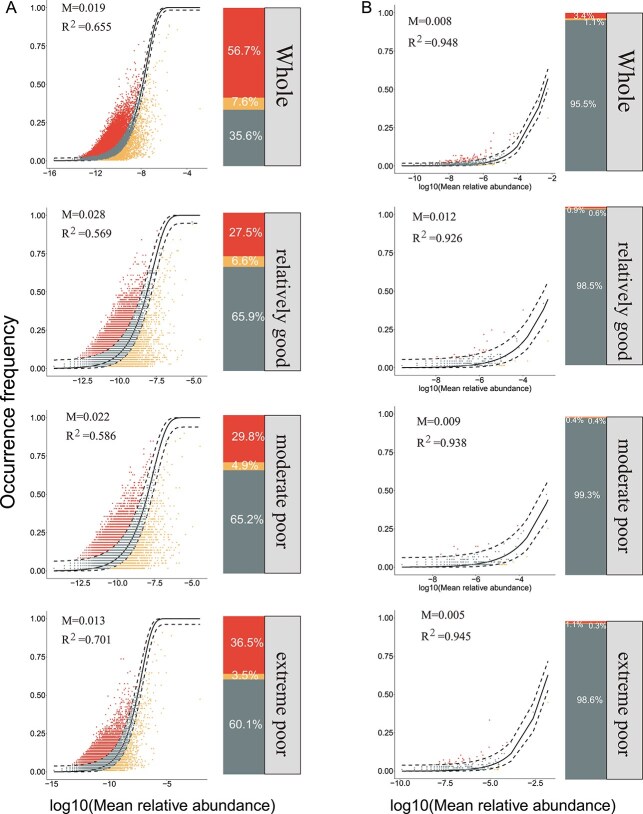
Neutral-model fit for bacterial (A) and fungal (B) communities. Each scatterplot shows the relationship between the metacommunity-wide mean relative abundance of every ZOTU (x-axis, log10-scaled) and its frequency of occurrence across samples (y-axis). Dots represent individual ZOTUs (amplicon sequence variants). The solid curve is the best neutral-model fit; dashed curves delineate the 95% confidence envelope of the model predictions. *R*^2^ quantifies the goodness of fit, and m is the estimated immigration (dispersal) rate. Below each scatterplot, a stacked bar chart summarizes the proportion of ZOTUs that are over-represented (points falling above the upper dashed line), neutrally distributed (points within the envelope) or under-represented (points below the lower dashed line) relative to neutral expectations. These proportions are expressed as percentages of the total sequences in the corresponding community type. Together, the scatterplots and bars illustrate the balance between stochastic (neutral) and deterministic processes in assembling bacterial vs fungal communities.

Overall, the frequency of bacterial ZOTU exhibited a moderate fit to the neutral model (*R*^2^ = 0.655). However, the model exhibited variations across different habitat qualities, with the order of fit being extreme poor (*R*^2^ = 0.701), moderate poor (*R*^2^ = 0.586), and relatively good (*R*^2^ = 0.569). The estimated mobility parameter (m) exhibited distinct patterns: relatively good environment (*m* = 0.028) > moderate poor environment (*m* = 0.022) > extreme poor environment (*m* = 0.013) (Fig. 3A). The relative abundances of ZOTUs in neutrally distributed bacterial communities were 65.9%, 65.2%, and 60.1% in the relatively good, moderate poor, and extreme poor environments, respectively (Fig. 3A). Fungi exhibited stronger adherence to the neutral model (*R*^2^ = 0.948) compared with the bacterial communities. The order of model variation across habitats of varying qualities is as follows: extreme poor (*R*^2^ = 0.945), moderate poor (*R*^2^ = 0.938), and relatively good (*R*^2^ = 0.926). In the neutrally distributed fungal communities of the three habitat qualities, the relative abundances of ZOTUs surpassed those of bacteria, constituting 98.5%, 99.3%, and 98.6%, respectively (Fig. 3B). Both the bacterial and fungal communities were strongly aligned with the neutral model. These results indicate that stochastic processes play a dominant role in the assembly of microbial communities in the Tarim River Basin. Spearman correlations revealed distinct responses of neutral taxa to habitat quality (RSEI) between bacteria and fungi (Fig. S5). For generalist communities, the relative abundance of neutral OTUs increased significantly with RSEI for both bacteria (ρ = 0.375, *P* = 5.24 × 10^−9^) and fungi (ρ = 0.199, *P* = 2.72 × 10^−3^), indicating that higher environmental quality favors stochastic processes and niche expansion in generalists (Fig. S5A and B). In contrast, specialist communities showed weak or opposite patterns: bacterial specialists were negatively correlated with RSEI (ρ = −0.137, *P* = .0398), while fungal specialists exhibited a weak positive correlation (ρ = 0.145, *P* = .0301) (Fig. S5C and D). These results suggest that improving habitat quality enhances the prevalence of neutral processes in generalist taxa, whereas specialists remain more influenced by deterministic environmental filtering, particularly under low-quality conditions.

**Figure 4:**
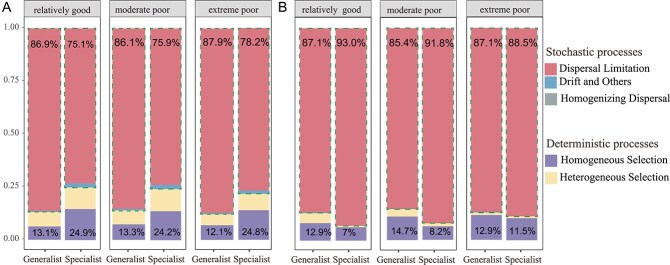
Community assembly processes of (A) bacteria and (B) fungi generalists and specialists. The deterministic processes include both homogeneous selection and heterogeneous selection, and the stochastic processes include homogenizing dispersal, dispersal limitation, drift, and others. The part enclosed by the dashed line represents the stochastic process. The numbers at the upper and lower ends of the bar chart represent the total contributions of deterministic and stochastic processes, respectively, to the community assembly.

The Sloan neutral model (Fig. 3) shows that stochastic processes remain important across habitats: for both bacteria and fungi, model fit (*R*^2^) decreases steadily with decreasing habitat quality. The iCAMP null model, which employs phylogenetic bins, was used to assess community assembly mechanisms and investigate ecological processes in microbial communities of varying qualities. Stochastic processes contributed to >80% of the generalist community in the three habitats and to >70% of the specialist community across the three habitats (Fig. 4A and B). As habitat quality deteriorated, the degree of conformity to the neutral model increased, as evidenced by the results of the null model analysis. Within the generalist bacterial community, the hierarchy of fit levels was as follows: extreme poor (87.9%) > relatively good (86.9%) > moderate poor (86.1%) (Fig. 4A). The increasing pattern was pronounced within the specialized bacterial community, with the following percentages: extreme poor environment (78.2%), moderate poor environment (75.9%), and relatively good environment (75.1%) (Fig. 4A). In both generalist and specialist communities, the percentage of stochastic processes exceeded 85% (Fig. 4). Like the results for generalist bacteria, the generalist fungal community exhibited a pattern of initial decline followed by an increase in habitat quality, ranking as relatively good environment (87.1%), moderate poor environment (85.4%), or extreme poor environment (87.1%) (Fig. 4B). In the fungal community, the prevalence of stochastic processes declined as habitat quality decreased (Fig. 4B). Furthermore, deterministic processes accounted for slightly higher proportions in bacterial specialist communities compared to generalists, whereas in fungal communities, generalists consistently exhibited marginally higher contributions from deterministic processes than specialists (Fig. 4).

### Identification of species contributing to differences in microbial communities

**Figure 5:**
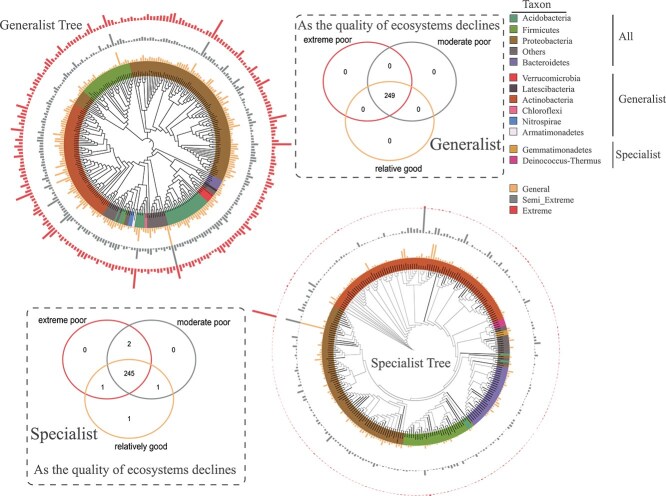
Phylogenetic tree and presence–absence patterns of the 250 ZOTUs identified by SIMPER analysis as contributing most to Bray–Curtis dissimilarity among habitat-quality classes. The Venn diagram shows the presence/absence of these SIMPER-selected taxa at the genus level in each habitat type; numbers refer only to this subset and do not indicate total richness or relative abundance. The phylogenetic tree includes bacterial genera represented by donut charts, with genus labels shown on the far right. The bar charts indicate the relative abundance of each genus within the three habitat types (relatively good, moderately poor, and extremely poor), as defined in the figure legend. This figure is intended to illustrate that many high-contribution taxa are detected in multiple habitats but differ markedly in relative abundance, highlighting abundance shifts rather than complete turnover as a key driver of compositional differences.

To pinpoint the taxa driving compositional differences among habitat-quality groups, we performed SIMPER analysis on the Bray–Curtis dissimilarity matrices of generalist and specialist communities separately. For each group, we selected the top 250 ZOTUs that contributed most to the observed dissimilarities across habitat types and visualized their distribution patterns (Fig. 5).

Within the bacterial community classified as generalists, many taxa in the top 250 were detected in all three habitats (Fig. 5A). In this subset, the relatively good environment contained one specialist ZOTU not found in the other two habitats, while the moderate poor and extreme poor environments together contained two unique specialist ZOTUs (Fig. 5B). Although Fig. 2 shows comparable phylum-level abundances of *Actinobacteria* and *Proteobacteria*, the SIMPER-selected top 250 ZOTUs are dominated by a *Pseudomonas* specialist, reflecting its disproportionate role in driving dissimilarity. Specialist *Actinobacteria* were represented by multiple genera, each contributing less individually and thus appearing more diffusely in the list. Phylogenetic analysis of these top 250 ZOTUs also revealed *Gemmatimonadetes* and *Deinococcus–Thermus* as exclusive specialists, whereas *Actinobacteria*, *Chloroflexi*, *Verrucomicrobia*, *Latescibacteria*, *Nitrospirae*, and *Armatimonadetes* appeared as generalists (Fig. 5).

Variations in fungal communities across the three habitat quality levels revealed the presence of 39 generalist ZOTUs common to all habitats, whereas the relatively good environment hosted a unique specialist ZOTU. The extreme poor and moderate poor habitats each harbored 10 ZOTUs shared with the relatively good habitat, as shown in Fig. S6A. The analysis identified 25 specialist ZOTUs that were shared among the three habitats, with the extreme poor environment exhibiting the greatest number of unique specialists (Fig. S6B). A phylogenetic tree was constructed to elucidate the species contributing to variations in fungal communities. Among the high-impact taxa, *Ascomycota* and *Basidiomycota* contained lineages classified as both generalists and specialists, whereas *Mortierellomycota* was represented exclusively by generalists (Fig. S6B).

### Specific associations between microbial community differences and physicochemical properties

**Figure 6:**
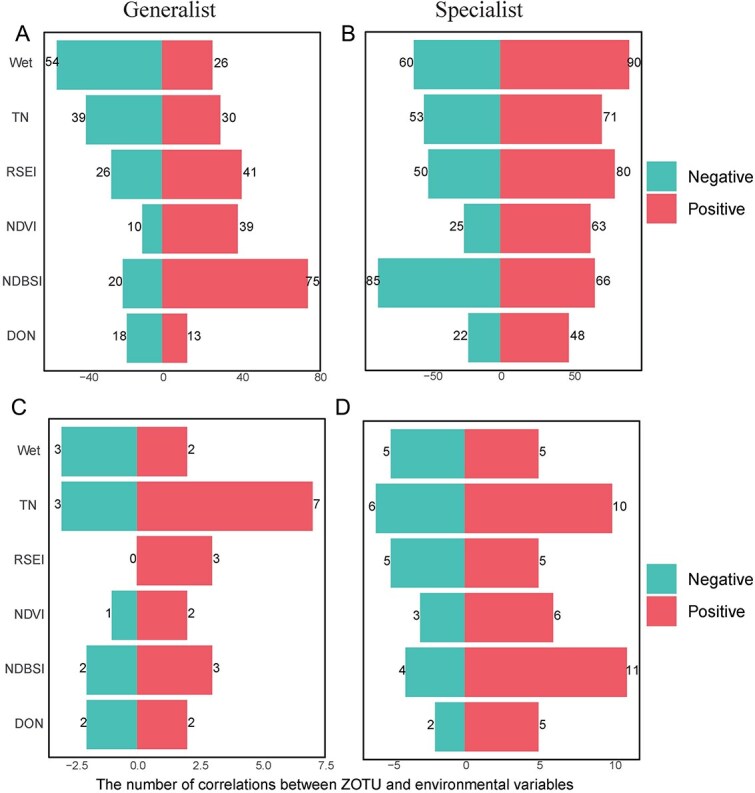
Number of significant correlations showing the associations between (A) bacterial and (B) fungal ZOTUs, physicochemical variables (TN, total nitrogen; DON, dissolved organic nitrogen), and climatic factors (RSEI, Remote Sensing Ecological Index; Wet, Normalized Difference Vegetation Index; NDVI, Normalized Difference Vegetation Index; NDBSI, Normalized Difference Built-up and Soil Index). Bar length indicates the number of significant correlations, and the direction of the bars indicates the sign of the correlations (negative or positive).

To investigate the environmental drivers of microbial community composition in the Tarim River Basin, we first analyzed the physicochemical properties of samples across the three habitat types (Fig. S7). Significant differences (*P* < .05) were observed among habitats in six key environmental variables: RSEI, total nitrogen (TN), normalized difference vegetation index (NDVI), soil moisture index (Wet), bare soil index (NDBSI), and dissolved organic nitrogen (DON). Analysis of the significant environmental factors and microbial communities revealed that RSEI and TN significantly affected the community structure changes of most species (Fig. S8). To further explore how these variables affect the microbial community, we conducted Spearman correlation analysis between the top 250 ZOTUs with the greatest differences in community composition and six environmental factors (Fig. 6). In bacterial communities (Fig. 6A and B), both generalists and specialists exhibited numerous significant associations with environmental variables. Notably, specialist taxa showed a higher total number of detected correlations, particularly with RSEI, TN, and NDVI. However, given that specialist ZOTUs are typically characterized by low abundance and restricted occurrence, their abundance matrices are inherently sparse and zero-inflated. As a result, rank-based correlation analyses may preferentially detect strong associations for specialist taxa. Therefore, the observed differences in correlation numbers are interpreted as differences in statistical detectability of environment-linked signals, rather than direct evidence of stronger biological responsiveness. Generalists, by contrast, showed the strongest correlation with NDBSI (75 positive, 20 negative), indicating sensitivity to surface brightness and likely land cover heterogeneity. In fungal communities (Fig. 6C and D), the overall number of significant correlations was markedly lower. However, fungal specialists again displayed a slightly broader response profile than generalists, with more frequent associations particularly positive ones with TN and NDBSI. For example, 11 specialist ZOTUs were positively correlated with NDBSI, compared to only 3 generalists. This pattern reinforces the notion that fungal specialists, while narrower in ecological amplitude, may exhibit strong sensitivity to specific environmental gradients, potentially due to reliance on stable microhabitats or specific resources.

## Discussion

Studying generalist and specialist communities in soils is essential for advancing research in environmental microbial ecology. We applied the RSEI as a holistic measure of environmental quality to capture the combined effects of multiple environmental variables on microbial communities, rather than examining each variable in isolation. This approach facilitates the study of diversity and community assembly of generalist and specialist communities in habitats under different environmental conditions. Moreover, this study differs from previous research that focused on the impact of individual environmental factors, overlooking the influence of interactions among multiple environmental variables on the diversity and composition of generalist and specialist communities within habitats.

Environmental changes shape microbial community composition and diversity, with bacteria and fungi responding differently. Bacterial communities varied significantly across habitats of differing quality, while fungal beta diversity remained stable (Fig. 1A), suggesting higher bacterial sensitivity to environmental fluctuations. This pattern reflects the strong spatial heterogeneity and environmental filtering typical of arid inland river systems [32]. In extreme poor habitats with low RSEI values, sharp variation in soil moisture, salinity, and organic matter are likely to increase environmental dissimilarity among sites, thereby enhancing beta-diversity [33]. By contrast, fungal beta-diversity remained relatively stable (Fig. 1B), suggesting that fungal communities possess higher tolerance or dispersal capability that buffers against local environmental heterogeneity [34]. These differences could stem from physiological and life-history traits: bacteria respond rapidly to moisture and nutrient pulses, while fungal hyphae and spores facilitate persistence across heterogeneous microhabitats [35]. The contrasting trends of generalist and specialist groups along the RSEI gradient (Fig. S2) further support the notion of niche differentiation under environmental stress [36, 37]. Elevated vegetation productivity (higher NDVI) may reduce competition by enhancing resource availability and spatial heterogeneity, facilitating microbial niche differentiation [38–40]. In relatively good, nutrient-rich environments, generalist taxa tend to dominate due to broader ecological tolerance. Conversely, in nutrient-poor, drought-stressed soils, specialists with narrow but efficient resource acquisition strategies dominate, consistent with stress dominance hypotheses observed in other desert systems [41, 42]. Consistent with these patterns, occupancy analyses (Fig. S3) further demonstrate that bacterial generalists occupy broader spatial ranges in habitats of moderate or high ecological quality, whereas specialists are more restricted and prevalent in low RSEI. This supports the notion that environmental filtering narrows bacterial niche breadth under increasing stress, favoring taxa adapted to specific microhabitats. In contrast, fungal occupancy showed minimal change across gradients, indicating that fungal taxa possess stronger physiological resilience and dispersal capacity, enabling persistence across heterogeneous or extreme environments. These findings reinforce that bacterial communities are more responsive to habitat quality, while fungi maintain compositional stability through structural and functional buffering.

Microbial community assembly in the Tarim River Basin was governed by both stochastic and deterministic processes, with stochasticity generally dominating (Figs 3 and 4). However, the balance between these forces varied along the environmental quality gradient, reflecting how extreme aridity and spatial heterogeneity modulate microbial turnover in this unique inland river ecosystem [41]. As habitat quality declined from temperate to extreme conditions, the relative influence of stochasticity decreased slightly (Fig. 4A), implying that environmental filtering becomes stronger under increasing stress. This transition is consistent with ecological theory predicting that harsher environments constrain viable niches and thereby strengthen deterministic control over community structure [36, 43]. The neutral model fitting (Fig. 3) revealed lower model fit (*R*^2^) and immigration rates (*M*) for bacteria than fungi, indicating that bacterial communities experience stronger environmental filtering and dispersal limitation, likely due to the high spatial patchiness of soil moisture and salinity across the Tarim floodplain [44]. In contrast, fungi showed higher stochasticity and dispersal-related processes, consistent with their hyphal connectivity and spore-mediated dispersal, which buffer against local environmental stress [45]. Across taxa, specialists were more strongly shaped by deterministic processes (especially homogeneous selection), reflecting their narrower ecological niches and higher sensitivity to environmental gradients such as total nitrogen and soil organic carbon [41]. This pattern is accentuated in the Tarim Basin, where soils transition from riparian zones with vegetation cover to barren desert crusts over short distances, generating steep microscale gradients in resource availability and salinity that intensify selective pressure [46]. Such environmental contrasts foster niche partitioning, explaining the higher beta-diversity observed among specialists. Interestingly, fungal generalists displayed stronger deterministic signals than specialists (Fig. 4B), potentially due to fungal symbioses with desert plants, where mycorrhizal associations create a consistent selection regime across microhabitats. These symbiotic interactions may override stochastic dispersal effects and impose stronger environmental filtering, a pattern also seen in other arid and semi-arid ecosystems [47–49].

To investigate the differences in microbial community composition between the generalist and specialist groups, phylogenetic trees were constructed (Figs. 5 and S6). The results of this study showed that there were more common ZOTUs in generalist bacterial and fungal communities in all habitat types (Figs. 5a and S6A). The broad ecological niche and wide range of environmental tolerances of generalist communities may explain this phenomenon [50]. *Gemmatimonadetes* and *Deinococcus–Thermus* were recognized as specialist bacterial communities, whereas *Rozellomycota*, *Entorrhizomycota*, and *Chytridiomycota* were identified as specialist fungal communities (Figs. 5b and S6B). *Gemmatimonadetes* and *Deinococcus–Thermus* exhibit notable resistance to radiation and thrive in diverse extreme environments, including those characterized by drought conditions [51, 52]. This observation aligns with previous research that identified *Gemmatimonadetes* as a minority population in coal mine sediments [53].

After identifying key taxa driving microbial community variation, we analyzed the correlation between environmental/climatic factors and the top 250 influential species. All indicators of habitat quality showed significant associations with both generalist and specialist communities (Fig. 6). Among them, RSEI and total nitrogen (TN) were the most consistently associated variables for bacterial community structure (Fig. 6A and B), aligning with prior studies highlighting nitrogen availability and ecosystem quality as major drivers of soil microbial diversity [54]. In this hyper-arid system, soil nitrogen strongly influences microbial turnover because nutrient inputs are spatially restricted to riparian corridors [55]. The tight coupling between TN and microbial composition is therefore indicative of pronounced biogeochemical filtering along environmental gradients. This pattern is in agreement with the stronger deterministic assembly signals inferred for specialist taxa based on null model analyses (Fig. 6), which reflect enhanced environmental filtering under constrained resource conditions. However, the higher number of significant environment taxon correlations detected for specialists should be interpreted with caution. Specialist taxa, by definition, occupy narrow ecological niches and occur in a limited subset of samples, resulting in sparse and zero-inflated abundance distributions. Under such conditions, correlation-based methods may preferentially detect sharp abundance shifts or presence–absence transitions along environmental gradients, thereby amplifying apparent associations [56]. Consequently, the observed enrichment of correlations among specialists likely reflects differences in statistical detectability rather than unequivocal evidence of stronger biological responsiveness. From an ecological perspective, this also suggests that correlation networks are inherently more sensitive to threshold-like responses characteristic of narrow-niche taxa, whereas the more gradual responses of generalists may be less readily captured by rank-based correlations [57]. Fungal communities also showed strong correlations with TN (Fig. 6C and D), consistent with findings in relatively good, alpine, and near urban ecosystems, where both generalist and specialist fungi respond to soil C and N availability [58, 59].

## Conclusion

This study employed RSEI to assess the ecological environmental quality of the Tarim River Basin and its influence on the microbial community. Instead of focusing on a single environmental factor, the analysis delved into the diversity of microbial communities (generalists and specialists), species composition, community assembly processes, and their association with environmental factors. The results revealed significant differences in the diversity between generalist and specialist microbial communities in habitats of varying quality. Stochastic processes predominantly influence microbial community assembly. The composition of generalists and specialists is influenced by the RSEI levels. Evolutionary traits indicate that generalists play key roles in maintaining taxonomic diversity. To further investigate the impact of habitat quality on ecosystem functions, the authors propose employing multi-omics approaches, such as metagenomics and metabolomics. This study enhances our understanding of how environmental quality shapes microbial diversity and distribution and provides novel methodologies and perspectives for ecological research.

## Supplementary Material

supplemental_material_ycag057

## Data Availability

The data that support the findings of this study have been deposited in the Sequence Read Archive (SRA) of NCBI under the BioProject accession number PRJNA1140827 and the BioProject accession number PRJNA1140882.
