# Supplementary material for: Environmental quality affects the formation of generalist and specialist taxa in microbial communities
Source: ISME Commun. 2026 Mar 17;6(1):ycag057. doi: 10.1093/ismeco/ycag057 (PMC13037473; doi:10.1093/ismeco/ycag057)
Supplement: supplemental_material_ycag057 [file supplemental_material_ycag057.docx]

Supplementary Materials

**This word file includes:**

**Supplementary Figures S1 to S6**

**Text S1**


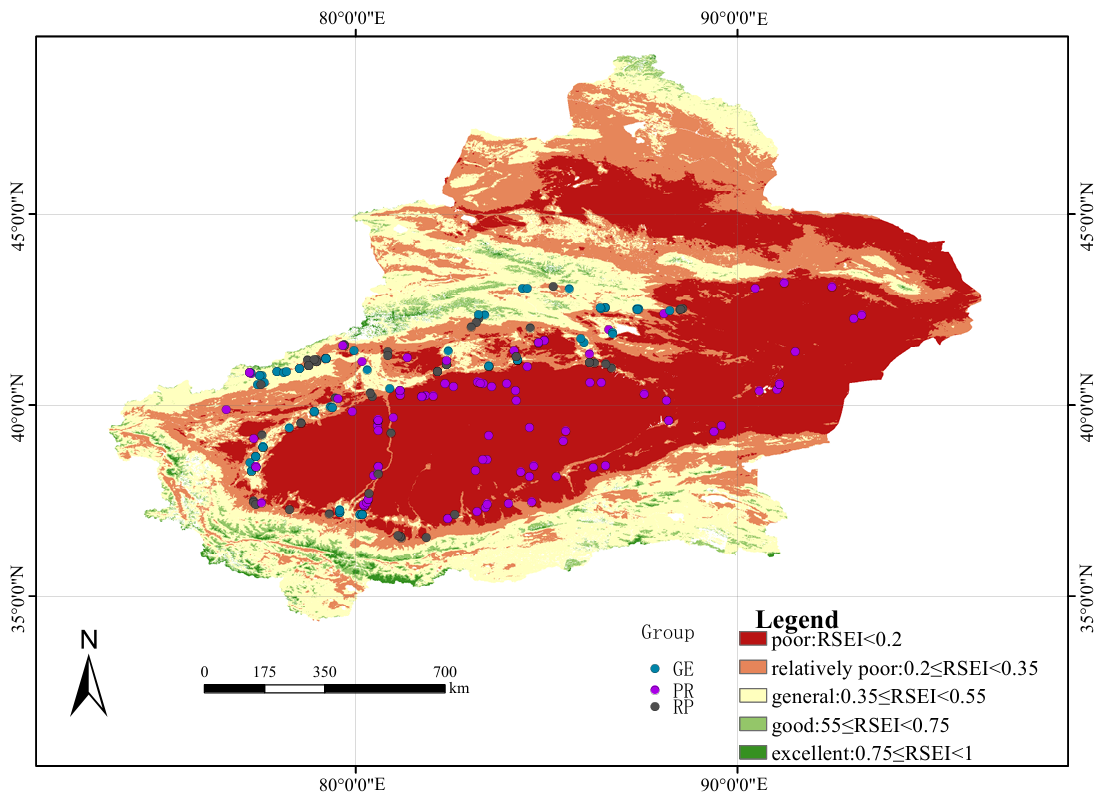


Fig S1. Sampling sites in the basin of the Tarim River, which is the largest inland river in China. Sites were classified into three habitat categories based on environmental quality:

GE: relative good (n = 69), RP: moderate poor (n = 59), and PR: extreme poor (n = 99).


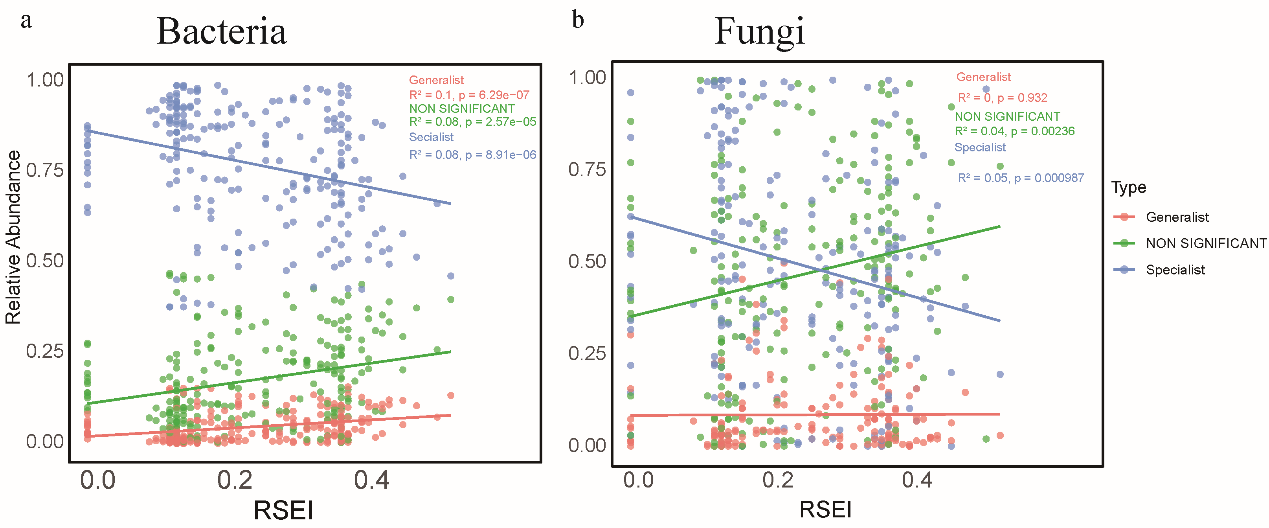


Fig S2. The linear regressions between the remote sensing–based ecological index (RSEI) and the relative abundance of generalist and specialist taxa revealed contrasting responses.


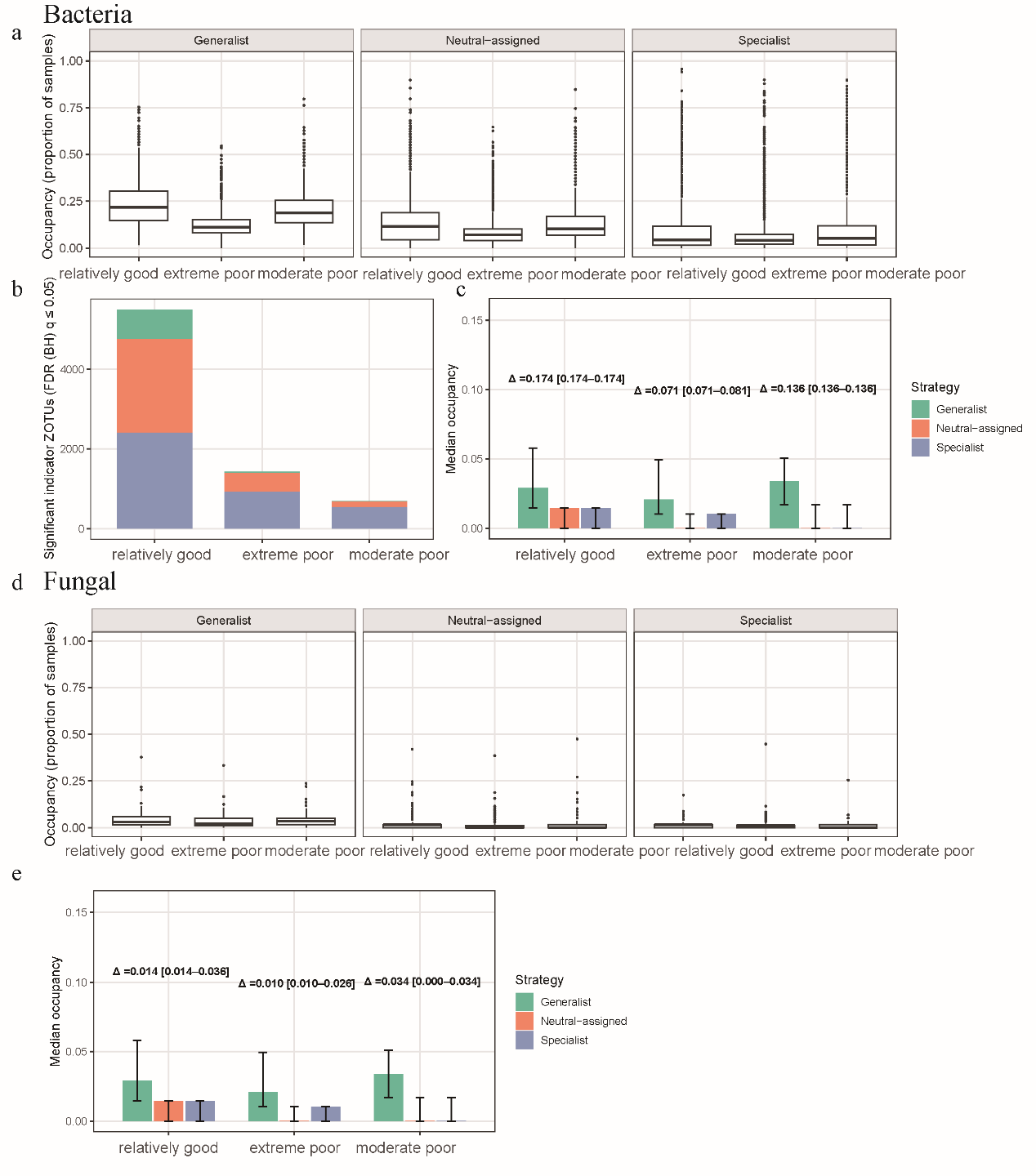


Fig S3. Occupancy breadth and habitat specificity by strategy across RSEI classes.
(a, d) Occupancy distributions (boxplots) for Generalist, Neutral-assigned, and Specialist ZOTUs within relative good/moderate poor/extreme poor environmental (occupancy = within-habitat proportion of samples with presence; threshold > 0 relative abundance). (b) Counts of significant indicator ZOTUs after BH–FDR control (q ≤ 0.05) per environmental quality, colored by strategy. (For fungi, no indicators remained after FDR.). (c, e) Median occupancy (bars) with IQR (error bars); numbers above bars denote Δmedian_{median}median​ (Generalist − Specialist) with 95% CI from 1000 bootstraps. “Neutral-assigned” denotes taxa not significantly broader or narrower than null expectations (Levins’ niche breadth with permutation CIs).


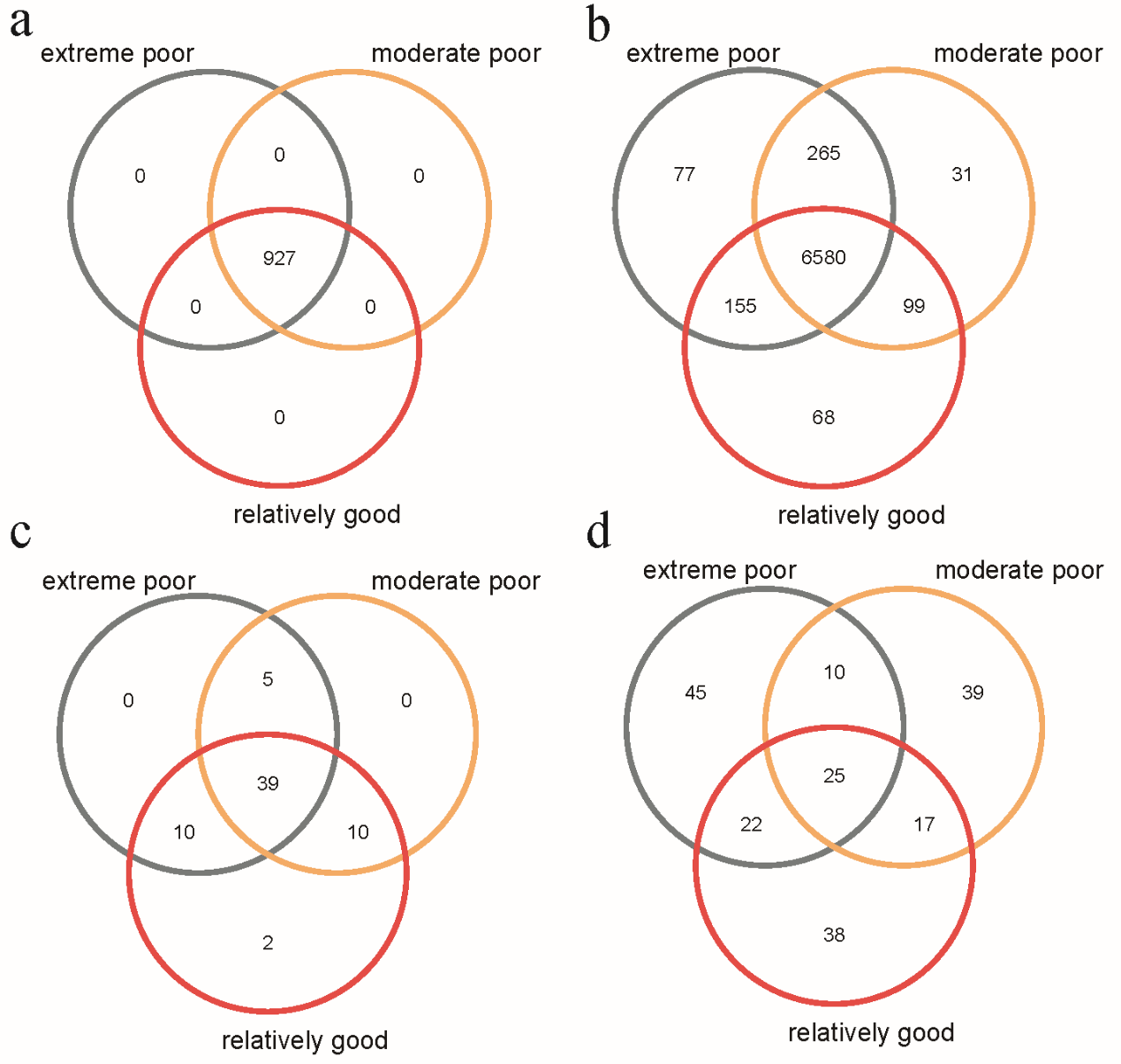


Fig S4. Venn diagram showing the overlap of ZOTUs between generalist/specialist environments. (a)bacterial generalist, (b)bacterial specialist, (c)fungal generalist, (d)fungal specialist.


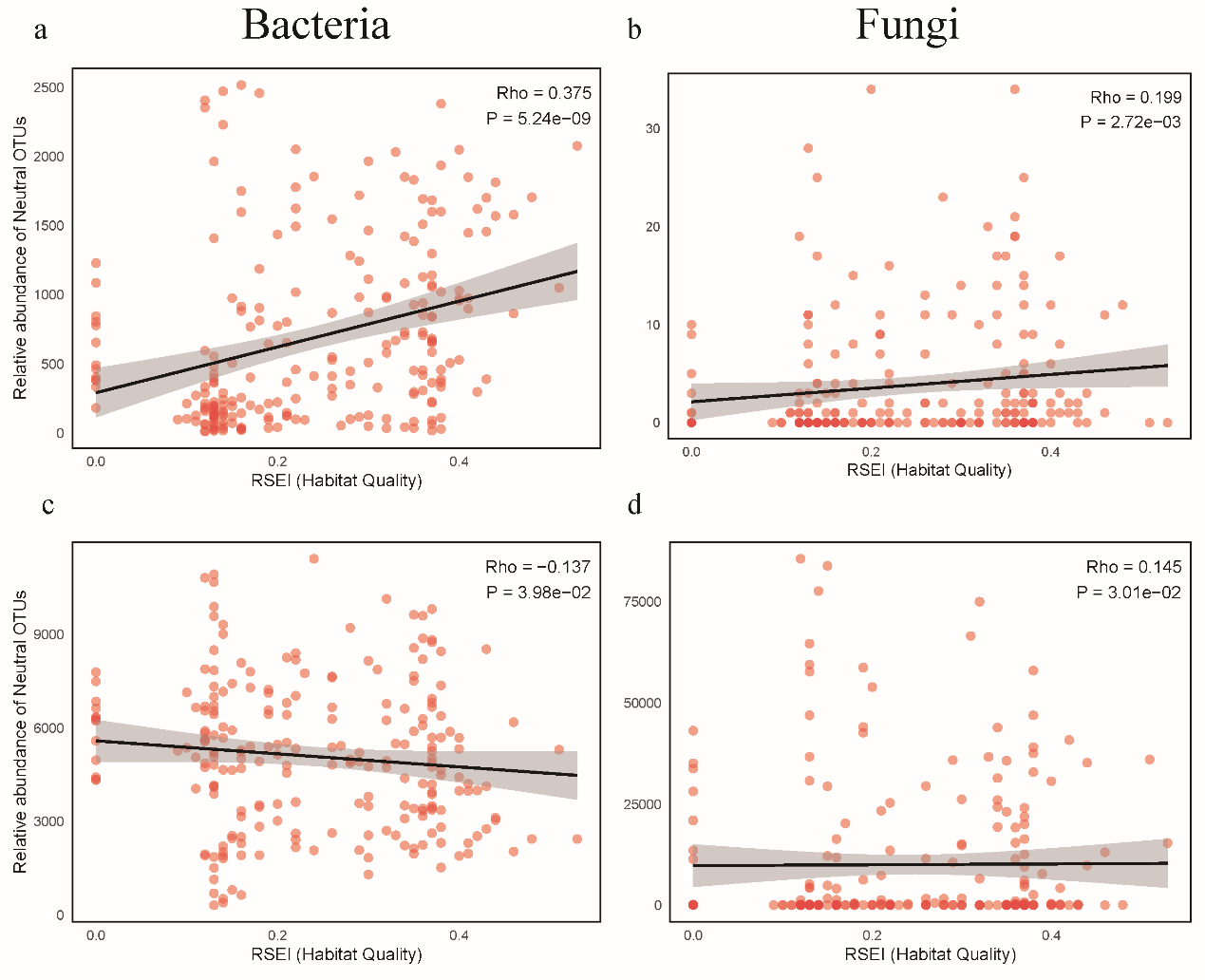


Fig S5. Relationship between habitat quality (RSEI) and the relative abundance of neutral OTUs in generalist and specialist communities. (a–b) bacterial and fungal generalist communities. (c-d) bacterial and fungal specialist communities. Rho and P values are based on Spearman’s correlations. Shaded areas represent 95% confidence intervals.


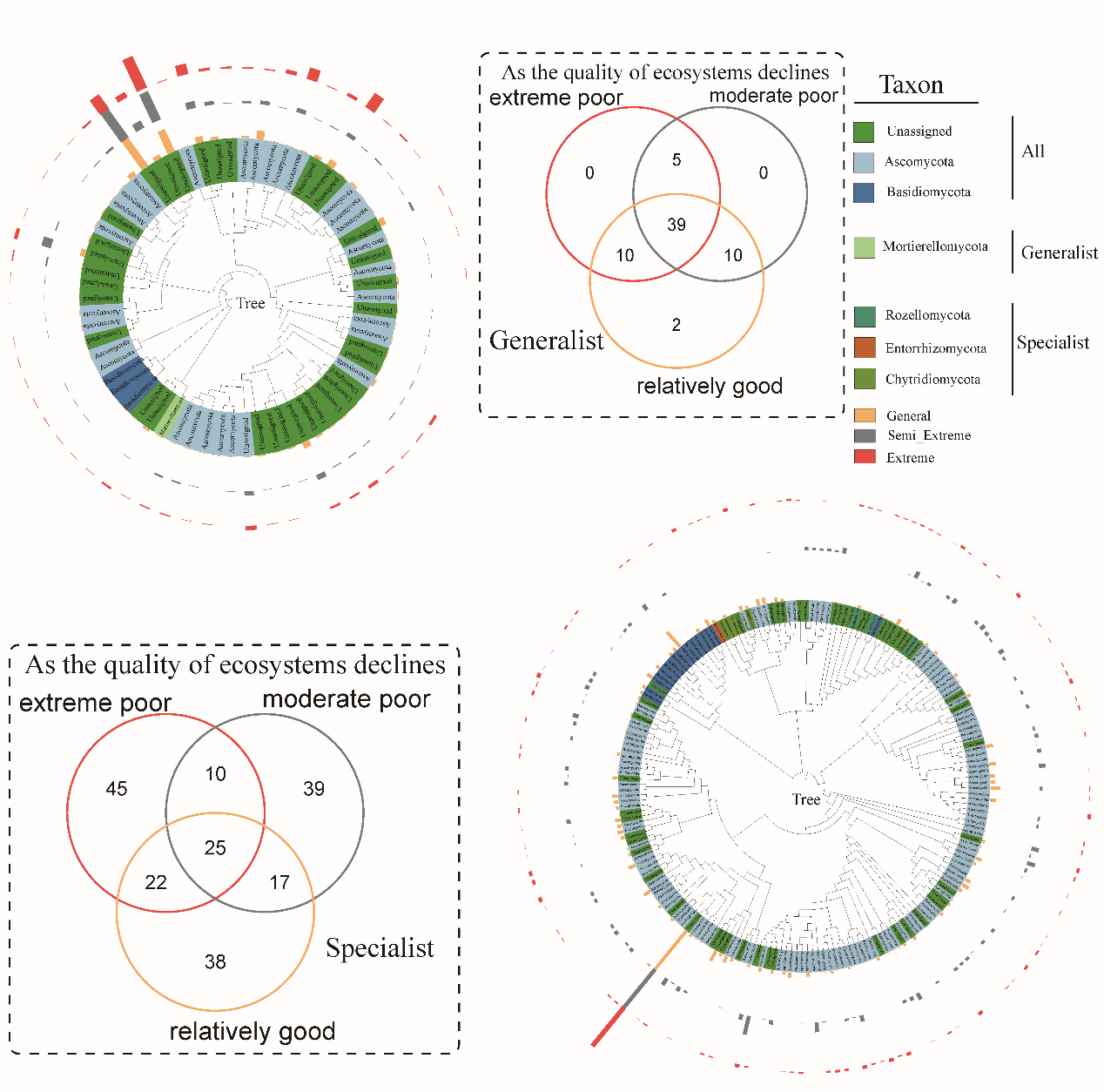


Fig S6. A phylogenetic tree was constructed on the basis of the greatest contribution to differences in fungal communities across various habitat qualities. The Venn diagram shows the genus-level differences in the fungal communities. The phylogenetic tree is shown in the middle of the figure, and the bacterial genus that make up the phylogenetic tree are arranged in donuts in different colors. The genus color label is shown on the far right. The bar chart shows the relative abundance of each species in different habitats, yellow: relative good, gray: moderate poor, red: extreme poor.


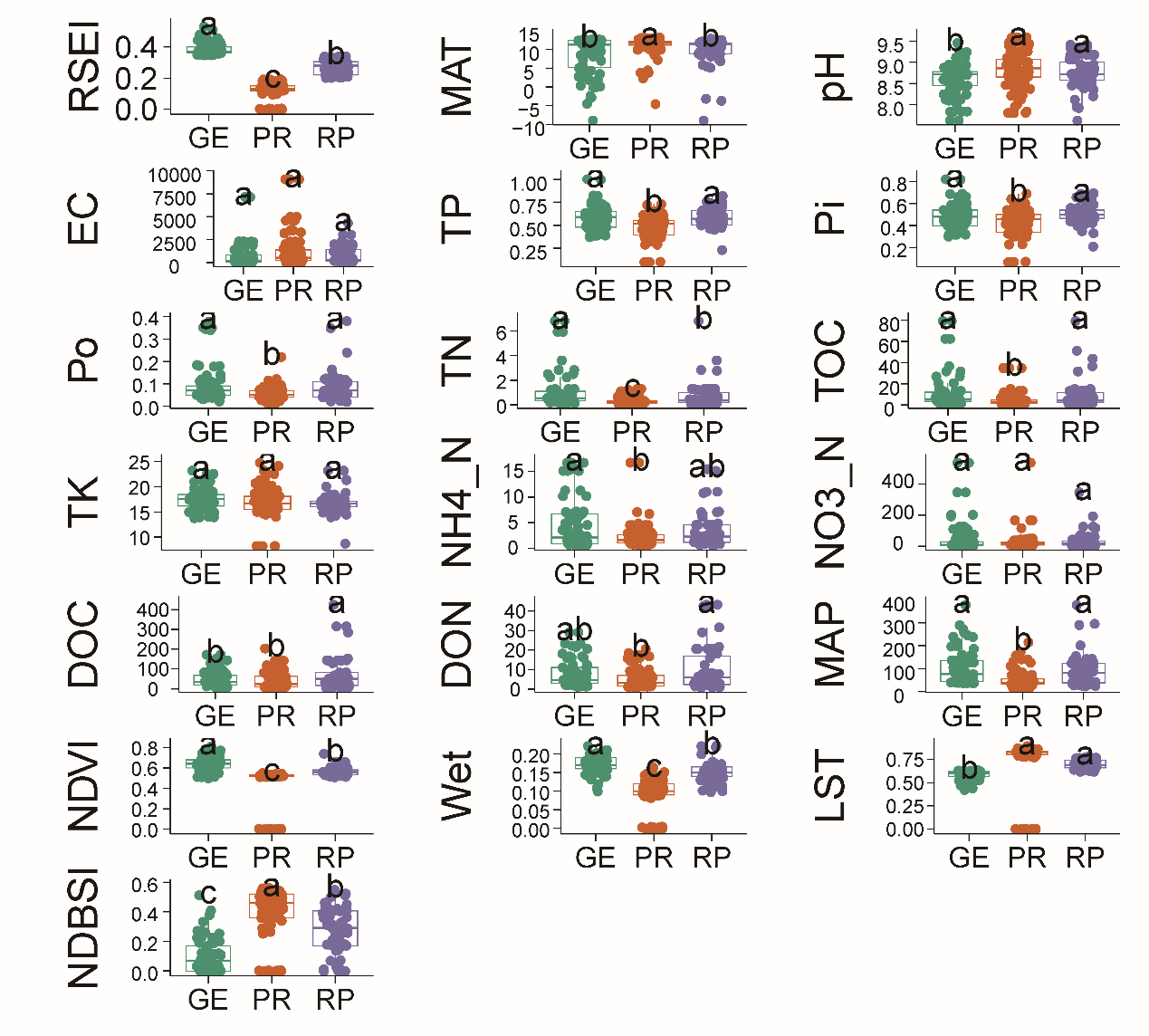


Fig S7. Environmental factors and climatic characteristics of sample sites. GE: relative good environmental, PR: Extreme poor environmental, RP: moderate poor environmental. total nitrogen (TN), total phosphorus (TP), total potassium (TK), ammonium nitrogen (NH_4_-N), nitrate nitrogen (NO_3_-N), total organic carbon (TOC), pH, Soil electrical conductivity (EC), soil organic phosphorus (SOP), inorganic P, Water-soluble organic carbon (DOC) and water-soluble organic nitrogen (DON), mean annual temperature (MAT) and mean annual precipitation (MAP).


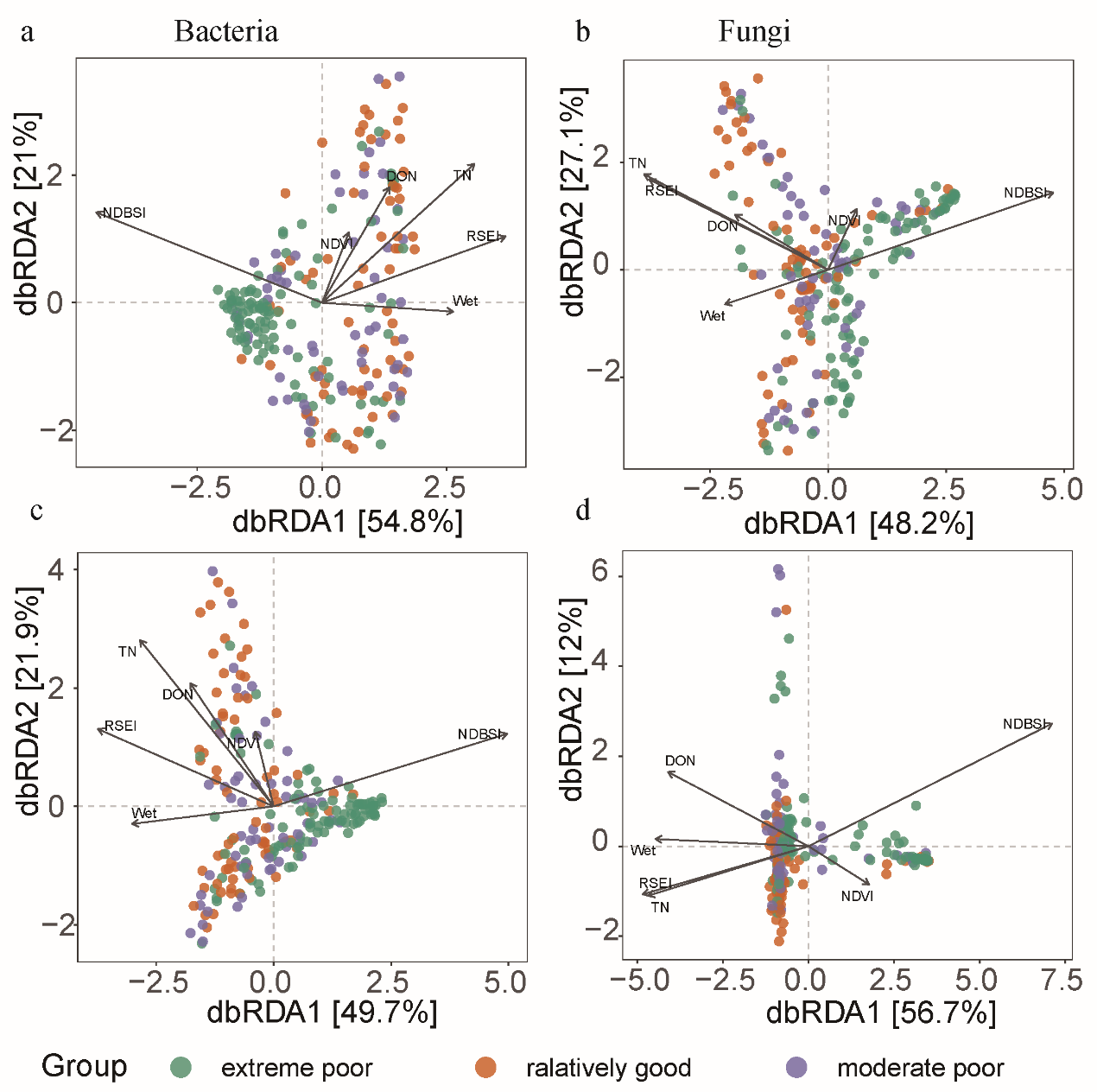


Fig S8. dbRDA analysis revealed that environmental factors drive the microbial community structure. (a-b) generalist, (c-d) specialist species. TN: total nitrogen, DON: dissolved organic nitrogen), and climatic factors (RSEI: Remote Sensing Ecological Index, Wet: Normalized Difference Vegetation Index, NDVI: Normalized Difference Vegetation Index, NDBSI: Normalized Difference Built-up and Soil Index.

Text S1

1.1 Study Area and Ecological Environment Classification

This study collected 227 soil samples from the Tarim River Basin in Xinjiang, China, covering an area of 102 × 10 km² with a geographic range of 34.20° N–43.39° N and 71.39° E–93.45° E. The information for all samples and the process of calculating the remote sensing-based ecological index (RSEI) at the sampling sites have been presented in a previous paper by the authors^[1]^ (Fig. S1). Briefly, the normalized difference vegetation index (NDVI), land surface moisture (Wet), land surface temperature (LST), and normalized differential built-up and bare soil index (NDBSI) were calculated as indicators of vegetation cover, humidity, temperature, and land use, respectively. After normalization, a principal component analysis (PCA) was performed, and the first principal component was used to calculate the Remote Sensing Ecological Index (RSEI). The resulting RSEI values were classified into five distinct habitat quality levels according to the Technical Criterion for Ecosystem Status Evaluation (HJ/T 192–2015) released by the Ministry of Ecology and Environment of China in 2015. For analytical clarity, these five levels were consolidated into three representative categories in this study: extreme poor, moderate poor, and relative good habitats. Field sampling was conducted from July to August 2022 at 227 stations across 102 × 104 km² of land in the Tarim River Basin. A 50 × 50 m quadrat was set up at each of the 227 stations. Within each quadrat, three soil cores (10–20 cm) were collected and combined to form a single sample representing each sampling site. A total of 227 samples were collected, with each sample weighing approximately 500 g. All samples were stored in cold storage at -4 °C. The sampling sites with RSEI values < 0.55 were divided into three habitat levels: extreme poor (N = 99, RSEI < 0.2), moderate poor (N = 59, 0.2 ≤ RSEI < 0.35), and relative good (N = 69, 0.35 ≤ RSEI < 0.55) environments.

1.2 Physical and chemical analyses of soil samples

The total nitrogen (TN) content was determined using the Kjeldahl method, while the total phosphorus (TP) content was determined using the NaOH melting-molybdenum antimony colorimetric method. The total potassium (TK) content was determined using the NaOH melting-flame photometer method, while the ammonium nitrogen (NH_4_-N) and nitrate nitrogen (NO_3_-N) contents were determined using the KCl leaching-indigo phenol blue colorimetric method. The total organic carbon (TOC) content was determined in accordance with Agricultural Chemical Analysis of Soil^[2]^; and the pH and electrical conductivity were measured using a benchtop acidimeter and benchtop conductivity meter, respectively. The soil’s organic phosphorus (SOP) and inorganic phosphorus (P) were determined using the Moore–Vandal method. Water-soluble organic carbon (DOC) and water-soluble organic nitrogen (DON) were determined using high-temperature catalytic oxidation. Climate data, such as the mean annual temperature (MAT) and mean annual precipitation (MAP), were obtained from WorldClim version 2 (https://www.worldclim.org) with a spatial resolution of 30 arcseconds.

1.3 DNA extraction and sequencing of microbial communities

Bacterial genomic DNA was extracted from the environmental samples using an Environmental DNA Extraction Kit (Omega Biotek, Norcross, GA, USA). The V3-V4 region of the bacterial 16S rRNA gene was amplified using the primer pair 338F (5′-ACTCCTACGGGAGGCAGCA-3′) and 806R (5′-GGACTACHVGGGTWTCTAAT-3′). Fungal sequencing of the ITS5 (5′-GGAAGTAAAAGTCGTAACAAGG-3′) and ITS2 (5′-GCTGGCGTTCTTCATCGATGC-3′) regions of ITS1(a) was performed. Amplicons were sequenced on an Illumina NovaSeq PE250 platform (Illumina, San Diego, CA, USA) using a paired-end sequencing model (PE250) at Personalbio, Nanjing, China.

1.4 Bioinformatics analysis

The original sequences were processed and analyzed using the workflows of USEARCH ^[3]^ (https://drive5.com/usearch/) and Vsearch^[4]^. Briefly, the barcode and primer sequences were stripped, the UNOISE3 algorithm in Usearch was used for denoising, and only the last 350 bases of the polymerase chain reaction (PCR) primers were selected for analysis (zero-radius operational taxonomic units (ZOTU)). High-quality sequences were grouped into operational taxonomic units based on 97% similarity. Chimeras were tested using Vsearch, bacteria were classified using the Ribosomal Database Project (RDP) classifier, and fungi were classified using the UNITE database. In total, 17,754,968 raw bacterial sequences were obtained from 227 samples. Dereplication was conducted using VSEARCH v2.22.1 (--derep_fulllength), and sequences occurring fewer than 20 times were removed using the --minuniquesize 20 parameter. This process retained 49,536 unique sequences for downstream analysis and discarded 9,544,173 low-abundance sequences (99.5%), which likely represented sequencing errors or spurious variants. After dereplication of 9,544,173 sequences, 49,536 were retained for analysis. Further noise reduction using the UNOISE3 algorithm of USEARCH identified 28,831 amplicons and generated 28,000 ZOTUs, while 831 sequences were identified as chimeras and were excluded. Similarly, 19,302,298 original fungal sequences were generated, which resulted in 1,456,935 unique sequences. After dereplication of1,417,530 sequences, 39,405 sequences were retained for analysis. After further noise reduction using the UNOISE3 algorithm of USEARCH, 8,267 amplicons were identified, resulting in 5,407 ZOTUs; 2,860 sequences were identified as chimeras and excluded. The minimum number of clean tags per bacterial and fungal sample was 49,536 and 39,405, respectively.

1.5 Calculation of Remote Sensing-Based Ecological Index

To calculate RSEI following Hanqiu Xu, we obtained four independent ecological indicators from MODIS data: greenness based on NDVI, humidity based on Wet, dryness based on NDBSI, and heat based on LST^[5]^. The RSEI is a function of these four indicators:

$$\begin{aligned} RSEI=f(NDVI,Wet,NDBSI,LST)\#\left( 1 \right) \end{aligned}$$

In the RSEI, the ecological significance of NDVI, Wet, NDBSI, and LST is integrated to produce a quantitative assessment of the ecological quality of the Tarim River Basin.

(1) Greenness index

The NDVI has been successfully used to monitor and assess vegetation cover across different scales. It has been used as an indicator of plant growth, vegetation density, and vegetation coverage^[6]^. It is defined as follows:

$$\begin{aligned} NDVI=\frac{\rho_{2}-\rho_{1}}{\rho_{2}+\rho_{1}}\#\left( 2 \right) \end{aligned}$$

where ρ_2_ and ρ_1_ represent the surface reflectance in the first (red) and second (near-infrared) bands of the MODIS data, respectively.

(2) Humidity index

Humidity can reflect vegetation and soil moisture content. We calculated the humidity index (Wet) using the tasseled cap transformation based on MODIS data following Zhang^[7]^:

$$\begin{aligned} Wet=0.1 147\rho_{1}+0.2 489\rho_{2}+0.2 408\rho_{3}+0.3 132\rho_{4}-0.3 122\rho_{5}+0.6 416\rho_{6}-0.5 087\rho_{7}\#\left( 3 \right) \end{aligned}$$

where ρᵢ (i=1,2,…,7) represents the surface reflectance of each of the seven bands in MODIS data.

(3) Dryness index

In this study, we constructed the dryness index based on MODIS data by averaging the new MODIS Building Index and the MODIS Bare Soil Index as follows:

$$\begin{aligned} NDBI_{M}=\frac{\rho_{1}-\rho_{4}}{\rho_{1}+\rho_{4}}\#\left( 4 \right) \end{aligned}$$

$$\begin{aligned} BSI_{M}=\frac{\left( \rho_{1}+\rho_{6} \right)-\left( \rho_{2}+\rho_{3} \right)}{\left( \rho_{1}+\rho_{6} \right)+\left( \rho_{2}+\rho_{3} \right)}\#\left( 5 \right) \end{aligned}$$

$$\begin{aligned} NDBSI=\frac{NDBI_{M}+BSI_{M}}{2}\#\left( 6 \right) \end{aligned}$$

where NDBI_M_ and BSI_M_ represent the MODIS Building Index and MODIS Bare Soil Index, respectively; ρᵢ (i=1,2,…,7) represents the surface reflectance of each of the seven bands in MODIS data.

(4) Heat index

The LST is an important component of the Earth’s energy budget and an important parameter representing the surface environment. The land surface temperature of the study area was extracted from the MOD11A2 land surface temperature product:

$$\begin{aligned} LST=0.02DN-273.15\#\left( 7 \right) \end{aligned}$$

where DN represents the pixel grayscale value of the MODIS land surface temperature data product and temperature values were converted from Kelvin to degrees Celsius.

(5) Construction of RSEI

The RSEI was constructed using the four indicators indicated above and PCA. To eliminate the influence of the scale of the different indicators on RSEI, the indicators were standardized, and values were compressed between 0 and 1:

$$\begin{aligned} X_{nom}=\frac{X-X_{min}}{X_{max}-X_{min}}\#\left( 8 \right) \end{aligned}$$

where Xnom is the standardized indicator, Xmin is the minimum value of the indicator, and Xmax is the maximum value of the indicator. The calculated initial Remote Sensing Ecological Index (RSEI_0_) was standardized to enable comparative analysis on the same scale:

$$\begin{aligned} RSEI={RSEI_{0}-RSEI_{min}}/{RSEI_{max}-RSEI_{min}}\#\left( 9 \right) \end{aligned}$$

where RSEI_min_ is the minimum value of RSEI and RSEI_max_ is the maximum value of RSEI.

**References**

[1] Yang G, Chen Y, Ren Q, Liu Q, Ren M, Zheng J, et al. Remote sensing ecological index (RSEI) affects microbial community diversity in ecosystems of different qualities[J]. Science of The Total Environment. 2024, 954: 176489.

[2] Tao H, Gemmer M, Bai Y, Su B, Mao W. Trends of streamflow in the Tarim River Basin during the past 50 years: Human impact or climate change?[J]. Journal of hydrology. 2011, 400(1-2): 1-9.

[3] Usearch E R. Lawrence Berkeley National Laboratory (LBNL)[J]. Berkeley, CA (United States). 2010.

[4] Rognes T, Flouri T, Nichols B, Quince C, Mahé F. VSEARCH: a versatile open source tool for metagenomics[J]. PeerJ. 2016, 4: e2584.

[5] 徐涵秋. 城市遥感生态指数的创建及其应用[J]. 生态学报. 2013, 33(24).

[6] Chen C, Fu J, Zhang S, Zhao X. Coastline information extraction based on the tasseled cap transformation of Landsat-8 OLI images[J]. Estuarine, Coastal and Shelf Science. 2019, 217: 281-291.

[7] Zhang X, Schaaf C B, Friedl M A, Strahler A H, Gao F, Hodges J C. MODIS tasseled cap transformation and its utility[C]. IEEE, 2002.
